# Reversible Brain Abnormalities in People Without Signs of Mountain Sickness During High-Altitude Exposure

**DOI:** 10.1038/srep33596

**Published:** 2016-09-16

**Authors:** Cunxiu Fan, Yuhua Zhao, Qian Yu, Wu Yin, Haipeng Liu, Jianzhong Lin, Tianhe Yang, Ming Fan, Luobu Gesang, Jiaxing Zhang

**Affiliations:** 1 Department of Physiology, Medical College of Xiamen University, Xiamen 361102, Fujian, China; 2 Institute of high altitude medicine, Tibet Autonomous Region People’s Hospital, Lasa 850000, Tibet Autonomous Region, China; 3 Department of Radiology, Tibet Autonomous Region People’s Hospital, Lasa 850000, Tibet Autonomous Region, China; 4 Magnetic Resonance Center, Zhongshan Hospital Xiamen University, Xiamen 361004, Fujian, China; 5 Department of Brain Protection and Plasticity, Institute of Basic Medical Sciences, Beijing 100850, China

## Abstract

A large proportion of lowlanders ascending to high-altitude (HA) show no signs of mountain sickness. Whether their brains have indeed suffered from HA environment and the persistent sequelae after return to lowland remain unknown. Thirty-one sea-level college students, who had a 30-day teaching on Qinghai-Tibet plateau underwent MRI scans before, during, and two months after HA exposure. Brain volume, cortical structures, and white matter microstructure were measured. Besides, serum neuron-specific enolase (NSE), C-reactive protein, and interleukin-6 and neuropsychiatric behaviors were tested. After 30-day HA exposure, the gray and white matter volumes and cortical surface areas significantly increased, with cortical thicknesses and curvatures changed in a wide spread regions; Anisotropy decreased with diffusivities increased in multiple sites of white matter tracts. Two months after HA exposure, cortical measurements returned to basal level. However, increased anisotropy with decreased diffusivities was observed. Behaviors and serum inflammatory factor did not significant changed during three time-point tests. NSE significantly decreased during HA but increased after HA exposure. Results suggest brain swelling occurred in people without neurological signs at HA, but no negative sequelae in cortical structures and neuropsychiatric functions were left after the return to lowlands. Reoxygenation changed white matter microstructure.

A large number of people move from lowlands to high-altitudes (HA) each year for leisure or work. Environmental factors at HA can affect physiological functions of the body. The brain is one of the organs in the body with the highest oxygen consumption and thus is particularly vulnerable to hypoxia^1^. In contrast, cold weather may result in hypothermia, which has been shown to have a neuroprotective effect against hypoxic encephalopathy^2^,^3^. Hypobaria can increase fluid permeability and induce white matter lesions^4^,^5^. After a period of HA living, people descend to lowlands and the altitude-acclimatized brain will probably suffer from the stresses generated by reoxygenation, hyperbaric atmosphere, and higher temperatures; and trigger different pathophysiological signs, leading to transient or irreversible tissue alterations.

Current neurologic data were mainly obtained from studies on mountain climbers ascending to extreme altitudes and developing acute mountain sickness (AMS). Among these climbers, some showed cerebral edema, microhemorrhage, cortical atrophy, and cortical and subcortical lesions^6^,^7^,^8^,^9^, while a small group did not display any changes in the brains^10^,^11^. Moreover, the lesions were still present in some of the affected climbers several months to years after their return to lowlands^6^,^7^,^9^,^12^.

In most cases, a large proportion of people, who moved from the lowlands to the HA regions did not show any neurologic symptoms. Whether their brains have suffered from the HA environment continues to be of significant concern; however, little has been shown in this field thus far. Our previous researches have focused on these individuals and employed magnetic resonance imaging (MRI)^13^,^14^. However, the previous two studies were case-comparison and small reports; moreover, the images were obtained several days after the subjects’ return to the lowlands and thus the brain structure could have been influenced by hypoxia/reoxygenation-induced changes of cerebral blood flow^15^. Furthermore, the subjects (mountain climbers and garrison of soldiers) were involved in intense physical activity, which could accentuate the effects of hypoxia^16^,^17^. Finally, there was no follow-up observation of the subject conducted, which could be considered as the most serious drawback of these two studies.

In the present study, 31 college students, who volunteered for 30 days teaching experience on the Qinghai-Tibet plateau, were recruited to investigate the brain structural changes after HA exposure and the possibility of persistent sequelae after their return to the lowlands. None of the subjects developed AMS during their stay on the plateau. Registration- and segmentation-based techniques based on MRI were used. Structural Image Evaluation, using Normalization, of Atrophy (SIENA). which measures temporal brain changes at different points in time, is one of the registration-based methods. It provides a global assessment of percentage brain volume change (PBVC). SIENA has been extended to a single time-point method (Structural Image Evaluation, Using Normalization, of Atrophy Cross-sectional, SIENAX)^18^, which provides global normalized brain volume (NBV), gray matter volume (GMV), and white matter volume (WMV). FreeSurfer, which measures cortical thickness, surface area, and curvature^19^, is one of the automatic segmentation-based algorithms. Both SIENA- and FreeSurfer-generated measures have advantages for GM volume analysis in post-processing algorithms over voxel-based morphometry (VBM)^20^,^21^ used in our previous studies^13^,^14^. In addition, Tract-Based Spatial Statistics (TBSS) approach was used to examine white matter (WM) fractional anisotropy (FA), mean diffusivity (MD), axial diffusivity (AD), and radial diffusivity (RD) which are associated with local cerebral edema^13^.

Glucose metabolism regulator neuron-specific enolase (NSE) can leak into the bloodstream after neuronal destruction^22^. HA hypoxia can promote inflammatory cytokines interleukin (IL)-6 and C-reactive protein (CRP)^23^. In the present study, serum NSE was measured to predict brain damages. We also measured serum IL-6 and CRP, as their upregulation could be responsible for the brain changes.

## Results

### Physiological characteristics

Lake Louise Score and body temperature were measured only during the subjects’ stay at the plateau. The maximum (1.8 ± 1.6) Lake Louise score was recorded for all of the subjects on the first day at HA, and with time, the scores gradually decreased to 0 before the subjects returned to sea level (SL). The body temperature gradually decreased from 36.7 ± 0.24 °C to 36.4 ± 0.33 °C. De-acclimatization symptoms scores were 8 in two subjects, 3 in three subjects, and <2 in the others.

In Test 2 compared with Test 1, heart rate, circulating erythrocyte count, hemoglobin level, and diastolic pressure were significantly increased, while SaO_2_ and pulmonary forced vital capacity (FVC) and forced expired volume in one second (FEV1) were significantly decreased (Table 1); circulating leukocytes (*p* = 0.002) and leukomonocytes (*p* < 0.001) were also significantly decreased. There were no significant differences between Test 3 and Test 1 in any of the measurements taken.

### Metabolic measurements

Compared with Test 1, serum NSE was significantly decreased in Test 2 (*p* < 0.001) and significantly increased in Test 3 (*p* = 0.016). There were no significant differences in serum IL-6 and CRP between the three tests.

### Neuropsychiatric characteristics

Neuropsychiatric characteristics of HA exposed college students are showed in Table 2. There were no significant differences in the scores of Beck Depression Inventory and Beck Anxiety Inventory between the tests at three time points. Although an increase of the behavioral scores in Wechsler Memory Scale subset tests (accumulation, figure memory, figure recognition, touch score, and Digit Span backward task) and the Rey-Osterrieth Comples Figure (ROCF) test during the tests at the three time points could be observed within both HA exposure and SL control groups, two-way repeated measures ANOVA did not discover any significant differences between the groups.

### Total brain volume

**Figure 1:**
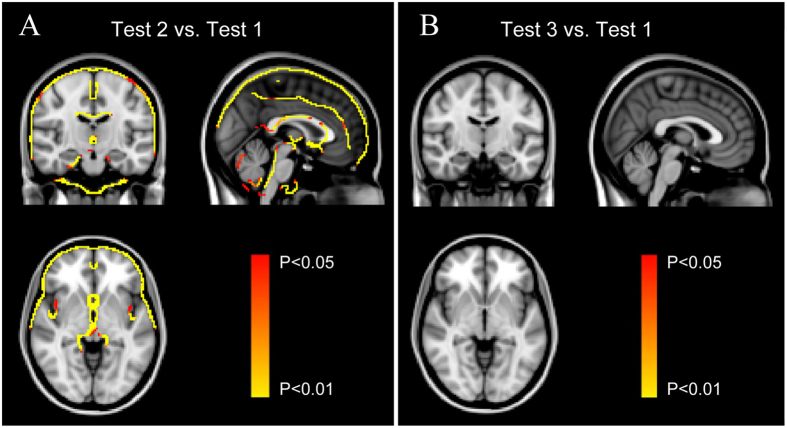
Significant morphometric edge flow indicating brain volume swelling during high altitude exposure (Test 2) (**A**) and two months after return to sea level (Test 3) (**B**) compared with baseline before ascent to high altitude (Test 1). Overlaid on MNI152 standard image. Voxelwise non-parametric SIENA statistics at *p* < 0.05 (corrected for multiple comparisons).

Compared with Test 1, there were markedly increased PBVC (2.6 ± 0.5%) and decreased cerebrospinal fluid (4.7 ± 0.8%) in Test 2. The enlarged regions in Test 2 included the bilateral inferior frontal gyrus, frontal pole, precentral gyrus, postcentral gyrus, lateral occipital cortex, temporal pole, paracingulate gyrus, and insula cortex as well as brain stem and multiple edges of cerebellum (Fig. 1A).

Compared with Test 1, there were slightly increased PBVC (0.4 ± 0.4%) and decreased cerebrospinal fluid (0.2 ± 0.5%) in Test 3, which did not show significant differences between the tests at these two time points (Fig. 1B).

**Figure 2:**
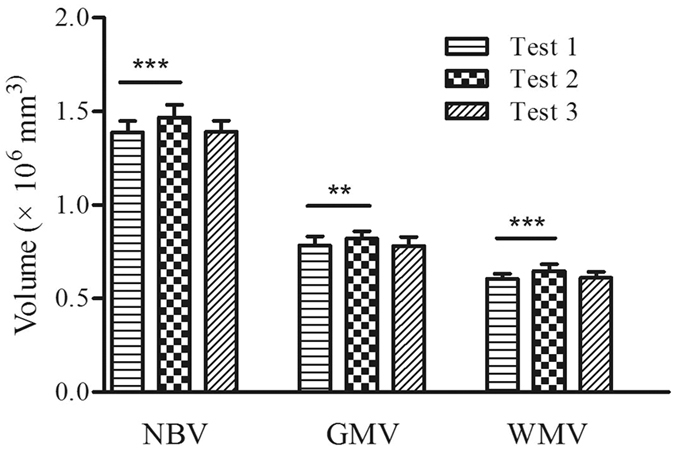
The normalized brain volume (NBV), gray matter volume (GMV), and white matter volume (WMV) measured before (Test 1), during (Test 2), and two months after (Test 3) HA exposure.

The NBV, GMV, and WMV were significantly increased in Test 2 compared with Test 1 (*p* < 0.001; *p* = 0.003; *p* < 0.001, respectively), but they have not showed any significant differences between Test 3 and Test 1 (Fig. 2).

### Cortical thickness, surface area, and curvature

**Figure 3:**
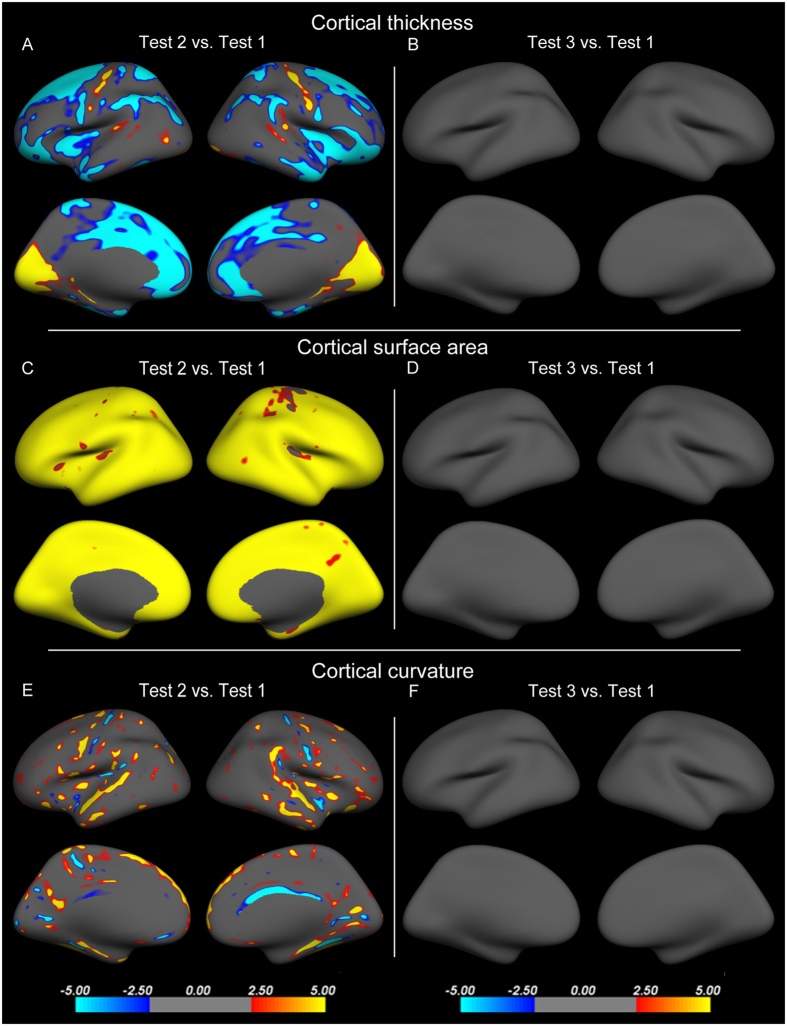
Cortical thickness, surface area, and curvature changes in subjects during high altitude exposure (Test 2) (**A,C,E**) and two months after return to sea level (Test 3) (**B,D,F**) compared with baseline before ascent to high altitude (Test 1). Yellow indicates an increase; blue indicates a decrease.

In Test 2, compared with Test 1, the cortical thickness (size > 350 mm^2^) was significantly decreased in the bilateral superior frontal gyrus, rostral anterior cingulate gyrus, superior parietal gyrus, supramarginal gyrus, and insula, left fusiform gyrus, and right inferiorparietal gyrus and increased in the bilateral pericalcarine gyrus and precentral gyrus (Fig. 3A; Table 3). Furthermore, the surface area was significantly increased across the whole cortices, except for the right precentral gyrus and bilateral posterior insula (Fig. 3C). Finally, the curvature was significantly increased in the bilateral precentral gyrus, superior frontal, supramarginal gyrus, inferior frontal gyrus, paracentral lobule, precuneus, superior parietal cortex, temporal gyrus, parahippocampal gyrus, insula, and fusiform gyrus and decreased in the postcentral gyrus and right cingulate gyrus (Fig. 3E).

In Test 3, compared with Test 1, no significant differences in cortical thickness, surface area, and curvature were detected (Fig. 3B,D,F).

### Correlations of cortical thickness and surface area with physiological measurements

**Figure 4:**
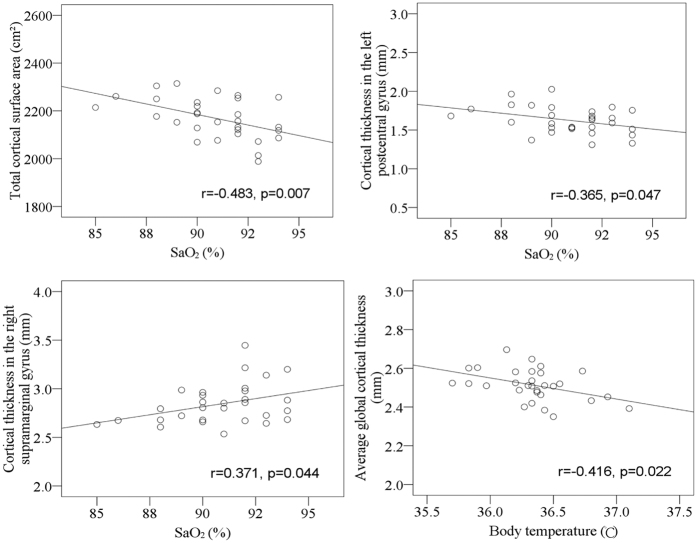
Correlations of cortical measurements with SaO_2_ and body temperature during high altitude exposure.

At HA, total cortical surface area and regional cortical thickness in the left postcentral gyrus was negatively correlated with SaO_2_, while cortical thickness in the right supramarginal gyrus was positively correlated with SaO_2_. Average global cortical thickness was negatively correlated with body temperature (Fig. 4).

**Figure 5:**
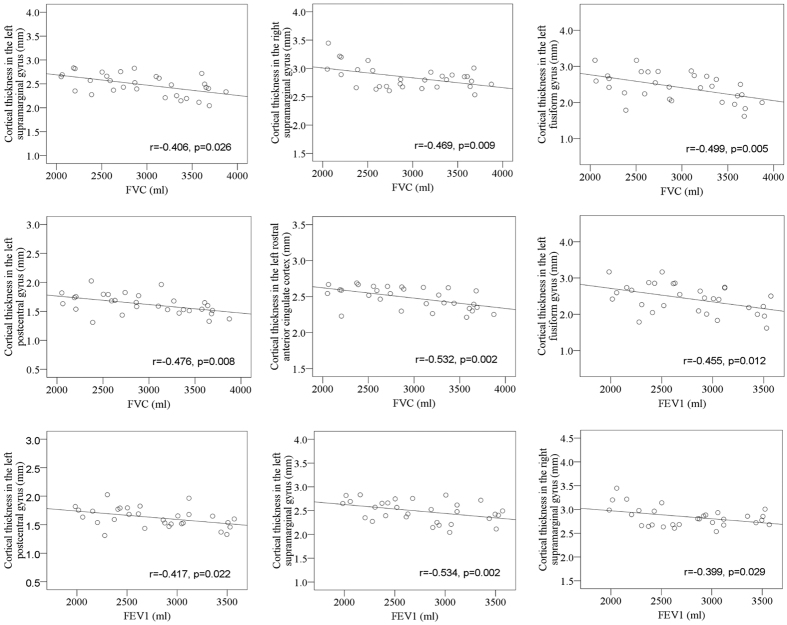
Correlations of regional cortical thickness with pulmonary variables during high altitude exposure.

At HA, cortical thickness in the bilateral supramarginal gyrus, left postcentral gyrus, and left fusiform gyrus was negatively correlated with both FVC and FEV1 and cortical thickness in the left rostral anterior cingulate cortex was negatively correlated with FVC (Fig. 5).

### FA and MD

**Figure 6:**
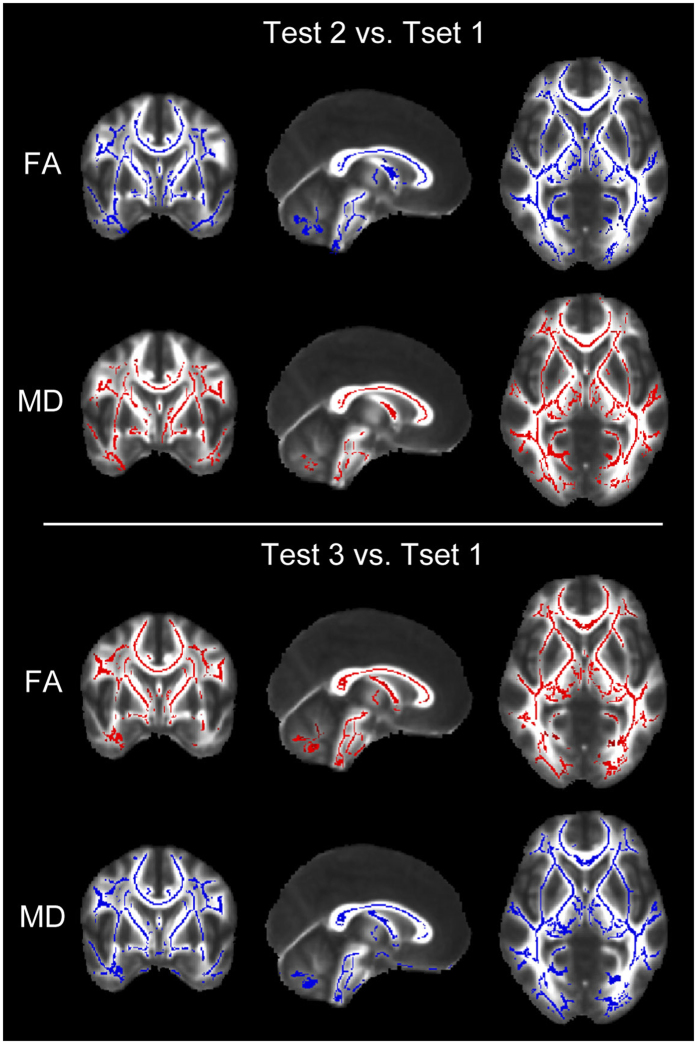
Statistical maps of comparison of fractional anisotropy (FA) and mean diffusivity (MD) values on a voxelwise basis. Compared with baseline Test 1, subjects showed significant lower FA value and higher MD during high altitude exposure (Test 2), while showed significant higher FA value and lower MD after return to sea level two months (Test 3). *p* < 0.001 (corrected for multiple comparisons). Red indicates an increase; blue indicates a decrease.

Compared with Test 1, in Test 2 the significant decreases of FA combined with increases of MD were detected in multiple sites of WM tracts (Fig. 6). In Test 2 vs. Test 1, the mean FA value was 0.436 (0.020) vs. 0.484 (0.026). *p* < 0.001; mean AD was 1.189 (0.030) vs. 1.145 (0.034), *p* < 0.001; mean RD was 0.575 (0.017) vs. 0.523 (0.048).

Conversely, the increases of FA with the decreases of MD were detected in multiple sites of WM tracts in Test 3 (Fig. 6). In Test 3 vs. Test 1, the mean FA value was 0.451 (0.016) vs. 0.400 (0.022). *p* < 0.001; mean AD was 0.870 (0.019) vs. 1.164 (0.032), *p* < 0.001; mean RD was 0.423 (0.011) vs. 0.645 (0.037).

## Discussion

The subjects in our study had low Lake Louise scores during the entire period spent at HA, indicating no experience of AMS. However, brain swelling was still found in both GM and WM. Further analysis found an enlarged cortical surface area across the whole brain as well as changed cortical thicknesses and curvatures. The increase of the total brain volume possibly reflects the extensive enlargement of the surface area rather than the changes of cortical thickness and curvature. Disrupted integrity of fiber tracts may have contributed to the increase of WM volume. In addition, HA exposure did not seem to affect individual neuropsychiatric function. Two month after the subjects returned to SL, all of the cortical measures returned to baseline values. However, unexpectedly, an increased FA with decreased axial and radial diffusivities was observed in the WM tracts. Serum NSE significantly decreased during HA exposure and subsequently increased at SL; however, this was not associated with cortical changes.

To our knowledge, this is the first ever study to look at the brain both before and after HA exposure, in which brain images were obtained at both the lowland and the extreme plateau altitude. In our study, 20 controls were also scanned and rescanned at an interval of 30 days and no differences in any cerebral measurements were found between the two MRI scans, which suggested that MRI instrument-related factors did not affect the morphological measurements. This is in-line with previous findings by Han *et al*.^24^ showing that the MRI scanner field strength, manufacturer, machine upgrade, and pulse sequence had little effect on reliability of cortical thickness measurements. The scan-rescan reliability of automated segmentation algorithms for cortical measurements was first confirmed by Morey *et al*.^25^. Later on, to assess the robustness of different post-processing algorithms applied to images acquired from different MRI systems, Durand-Dubief *et al*.^26^ scanned patients with multiple sclerosis over one year (at three time-points) on Intera and Sonata systems, using different sequences, and only small differences of 0.07% and 0.79% between the two systems were shown for the FreeSurfer and SIENAX analyses, respectively. Reuter *et al*.^27^ suggested that FreeSurfer could be a useful tool for the investigation of longitudinal brain development and pathophysiological changes. Based on the results of those experiments, a reasonable assumption of reliability for scans at one- to two-month intervals on two different scanner platforms was made.

Increased extent of vasogenic edema can significantly decrease FA and increase RD and AD^28^. Therefore, our results suggest vasogenic edema occurred in the WM during HA exposure, which may have contributed to the increase of the WM volume. Our findings were consistent with the results from several previous studies on hypoxic/ischemic brains^8^,^29^. Hypoxia-induced regional changes in autoregulation, cerebral blood flow, and cerebral capillary pressure may be sufficient to produce vasogenic edema^8^. However, with time spent at HA, the Lake Louise scores gradually decreased, suggesting brain edema was not associated with AMS.

The increases of FA with lower MD in WM tracts two months after return to SL were consistent with our previous observations in young HA Han residents one to three years after descent to the low altitude^30^ and in HA native Tibetan adolescents four years after descent to the lowlands^31^. A study conducted by Hackett *et al*.^8^ showed that patients with AMS recovered from edema six weeks to 11 months after return to SL. However, the report did not provide more details on microstructural characteristic of the WM tracts. Simultaneous increase of FA and decreases of AD and RD found in our study were consistent with the findings in children during their first year of life^32^,^33^, which were explained with fiber organization and axonal myelination^33^. However, for young adults in our study, these changed DTI scalars should not be interpreted globally as “good” or “bad”. Previous studies showed increase of FA as potentially reflecting both compensatory mechanisms^34^ and poor cognitive functioning^35^. In our study, the gradually enhance of behavioral performances during the tests at the three time points may attribute to learning effect. Moreover, increase of FA and decreases of MD, AD, and RD have been found in burning mouth syndrome^36^. Neurogenesis induced by low-to-moderate level hyperoxia has been proved *in vitro* and vivo observations^37^ and cytoskeletal α-tubulin and β-tubulin levels were strongly increased after hypobaric hypoxia/reoxygenation^38^. Therefore, the proliferation of glia and intracellular compartments of neuronal axons may be associated with a decrease in RD and AD^33^. Definitive conclusions about these DTI scalars of WM can only be derived from direct microscopic examination of brain tissue in the future animal study.

The increases of GM at HA may be associated with hypoxia-induced gliogenesis. Glial cells comprise more than 85% of the total population of brain cells. They are sensitive to changes in oxygen partial pressure^39^ and can be activated by hypoxia^40^. The decreases of cortical GM may be due to the neuronal loss^41^, but it seems that this is not the case, as no related behavioral disorders occurred. Vasculature accounts for about 5% of GM^42^. The capillary length per unit volume of tissue, dilation, and density in cortex increased after three weeks of hypoxia exposure, while the hypoxia-induced increase of blood flow has returned to baseline level^43^,^44^. Furthermore, our study detected high blood pressure during HA exposure, which could lead to thickening and hardening of the walls of arterioles and narrowing of the lumen, resulting in cerebral hypoperfusion^45^,^46^. Therefore, unbalanced development between angiogenesis and reduced cerebral blood flow could also determine regional cortical thickness. Previous reports have shown the cortical lesions persisted for more than several months after brief episodes of mountain climbing^7^,^12^. However, in our study, measured parameters in cortical GM have reverted back to baseline within two months of the subjects’ return to SL. The mechanism underlying this reversibility is likely due to reoxygenation reversing the increased capillary density, observed in hypoxia, to normoxic values^47^,^48^.

In the present study, alterations in GM volume (thickness and surface area) at HA were identified in the anterior insular cortex, anterior cingulate cortex, dorsolateral prefrontal cortex, supplementary motor area, posterior parietal cortex, supramarginal gyrus, and fusiform gyrus and the cortical thickness in most of these regions was significantly correlated with the FVC and FEV1, which is evidence that these regions play an important role in respiratory control and perception^49^,^50^,^51^. Furthermore, the decrease of thicknesses in the anterior insula and anterior cingulate cortex may be associated with the increased heart rate and blood pressure, as lesions of the right posterior insula increased baseline heart rate and blood pressure, electrical stimulation of the left insula of awake epileptic patients produced bradycardia, and decrease of neuronal activity in the right anterior cingulate cortex was correlated with higher heart rate^52^. The increase of cortical thickness in visual cortex, which has also been found in our previous study on adults who immigrated to the Qinghai-Tibet Plateau (2300–4400 m) for 2 years^14^, may be a compensatory mechanism to overcome the damage of the visual function caused by ultraviolet radiation at HA^53^. The changes in the cortical GM of particular brain regions observed in our present study had been previously found in patients with obstructive sleep apnea and in our previous studies in subjects exposed to HA^14^,^30^.

Hypoxia, hypobaria, and cold can exert their effects together on the brain at HA. Cerebral edema has been shown to occur after acute exposure to hypoxia in various normobaric conditions^29^,^54^,^55^, indicating the changes of the brain structures could be induced by the hypoxia alone. Our results support this suggestion, showing direct correlations of SaO_2_ with cortical measurements. However, several studies have shown that AMS scores were higher^56^,^57^,^58^, while visual sensitivity was lower^59^ in hypobaric hypoxia than in normobaric hypoxia, suggesting hypobaria at HA could also be a factor contributing to the brain lesions. WM lesions and the aggravation of depression-like behavior have been reported after exposure to nonhypoxic hypobaria^5^,^60^. The temperature at HA fluctuated between 4–17 °C, which was far lower than that in Xiamen (25–33 °C), and thus led to a gradual decrease of the body temperature. This hypothermia is considered neuroprotective in cerebral hypoxia^2^,^3^. Moreover, across all tests in this study, no significant changes in IL-6 and CRP were found, suggesting inflammation may not contribute to the structural changes of the brain.

In support of our findings, gradual increases of serum NSE were found in 613 soldiers two to 15 days after return to the lowlands from a 116 days stay at HA (3700 m)^61^ and in railway construction workers 19–66 months after return to the lowlands from six- to 60-month works at the Qinghai-Tibet plateau (3080–5072 m)^62^. Gradual increase of NSE expression, which peaked after five days of reoxygenation, has also been detected in rat brain during an experiment involving hypoxia/reoxygenation^22^. NSE is not only expressed in the neurons, but also in the peripheral neuroendocrine tissues and in amine precursor uptake cells^63^. In the present study, no direct correlations between serum NSE and brain structural measurements or de-acclimatization score (partially reflects brain function) were identified, suggesting the changes of serum NSE may result from oxygen-induced abnormal metabolisms in both neuronal and non-neuronal cells. The NSE level in the cerebrospinal fluid can be employed as a direct indication of neuronal damage, however it was not feasible in our study.

There were several limitations in our present study. One limitation was that the lifestyle at the plateau was different to the one the subjects were used to, for example in the absence of crowds, which could have affected the subjects’ emotional well-being. However, the subjects did not show obvious signs of depression or anxiety before their descent to the lowlands. In addition, subjects could also be challenged by the cultural change. Diet was not likely to have been an important factor for the observed brain changes, because the food in Xiamen and at the Qinghai-Tibetan Plateau was similar and the subjects were able to eat without any significant alterations to their dietary habits.

In summary, this is the first, longitudinal, detailed investigations of the total cerebral volume, cortical GM, and subcortical WM at the time of a stay at HA and after the return to the lowlands. Vasogenic edema at HA may be attributed to the increase of WM volume, while the mechanisms underlying the changes of GM volume were speculative. The cerebral cortices changed in the regions associated with cardiovascular and respiratory regulations. The cerebral effects of HA hypoxic exposure were reversible. However, reoxygenation at the lowlands simultaneously increased fractional anisotropy and decreased diffusivities in WM. Future studies should be conducted in animals to verify these findings and to clarify the mechanisms. Moreover, it seems no neuropsychiatric sequelae accompanied the brain structural changes.

## Methods

### Participants

The subjects were 31 healthy college students (16 men and 15 women, average age 19.7 ± 0.7 years) from Xiamen University in Xiamen (China). They took part in a 30-day teaching experience as volunteer teachers on Tibetan plateau during summer holidays in August, 2014. They were lowlanders, born and living at lowlands (<500 m), without any prior exposure to HA. All subjects had normal body mass index. The whole group had successfully finished teaching work, without the use of supplementary oxygen. Subjects were excluded if they developed mountain sickness during their teaching period, had a documented neurological disorder, or had a history of head injury. Another 20 healthy college students of comparable age, gender, and educational background, were recruited from Xiamen University as controls for verification of the reliability of the cerebral measurements that could be affected by MRI instrument-related factors, including scan-rescan using the same or different sequences; and as controls in the behavioral test performed at three time-points. These control students were tested in Xiamen. Procedures were fully explained and all subjects provided written, informed consent before participating in the study. The experimental protocol was approved by the Research Ethics Review Board of Xiamen University. All experiments were carried out in accordance with the approved guidelines.

### Plateau trip and experimental design

During the first three days, the subjects left Xiamen (sea level, SL) for Lasa (3650 m, Tibet, China). After four-day stay at Lasa, the subjects spent four hours travelling to Dangxiong city (4300 m, Tibet). On the 29th day, they finished teaching work and descended to Lasa. Four days later, they returned to Xiamen. At Dangxiong, the subjects had access to similar food and drink as that in Xiamen. No subject was a smoker and was allowed access to alcohol. During the entire period, the subjects stayed only at 4300 m.

A baseline set of sea-level physiological and neuropsychiatric tests, metabolic measurements, and MR images, were initially acquired in Xiamen before ascent to HA (Test 1); the same set of tests was performed at the plateau one to four days before the descent to Xiamen (Test 2); the final set of tests was performed after the participants had been living at SL again for two months (Test 3).

### Physiological measurements

Physiological tests included heart rate, blood pressure, hematological measure, arterial oxygen saturation (SaO_2_), and pulmonary function measure. Blood samples were taken in the morning between 07:00 and 07:30 h. The measurements at HA also consisted of the daily observations of the body temperature and the Lake Louise score, with a score greater than 4 being defined as AMS^58^. Axillary temperature and Lake Louise score were measured on the days between subjects arriving at Lasa and descending to Lasa in the afternoon between 19:00 and 19:30 h. Moreover, de-acclimation was tested within three days of subjects returning to SL. Symptom score and diagnostic criteria for de-acclimatization syndrome were adopted from He *et al*.^64^.

### Neuropsychiatric tests

Subjects were given the following tests: (1) The Chinese revised version of Wechsler Memory Scale provided measures of new verbal learning as well as visual and verbal memory functions. (2) The Serial Reaction Time Task (SRTT) was used to measure simple visuomotor implicit procedural learning. (3) ROCF assessed short- and long-term visual memory and visuoconstructional ability. The procedures of the above tests have been described in our previous study^14^. (4) The score of the Purdue pegboard test was measured by calculating the number of pins that the subject was able to place in the holes in 30 seconds^65^. The test involved gross movements of arms, hands, and fingers, and fine motor control. Poor Pegboard performance is a sign of deficits in complex, visually guided, or coordinated movements. (5) In addition, all subjects completed the Beck Depression Inventory and Beck Anxiety Inventory, which assessed the severity of depression and anxiety.

### Metabolic measurements

Serum NSE and IL-6 were measured by electrochemiluminescence immunoassay (ECLIA) on the Roche MODULAR ANALYTICS E170 (Elecsys module) immunoassay analyzer (NSE, Roche Diagnostics GmbH, D-68298 Mannheim). The sensitivity of the assay was <0.05 ng/ml. The inter- and intra-assay coefficients of variation were 3.8% and 1.6% respectively. High-sensitivity serum CRP was measured with particle enhanced immunonephelometry using BN-II system Nephelometer (Dade-Behring, Marburg/Germany), with a detection limit of 0.159 mg/L.

### Data analysis of physiological, neuropsychiatric, and metabolic measurements

Paired samples t-test was applied to analyze the differences between the tests performed at the three time-points. Differences in the results of the behavioral test over the three time-points between the experimental group and the control group were analyzed using two-way ANOVA with repeated measures. SPSS 16.0 software was used for data analysis. Statistical significance was set at *p* < 0.05.

### MRI data acquisition

The brain images were acquired on the same model of the Tim Trio 3T scanner (Siemens, Erlangen, Germany) at the MRI Center in Xiamen (Zhongshan Hospital, Xiamen, China) and at the MRI Center in Lasa (Tibet Autonomous Region People’s Hospital, Lasa, China). A 3D structural MRI was acquired using a T1-weighted MPRAGE sequence (TR/TE = 1900/2.7 ms, FOV = 25 × 25 cm^2^, average = 1, matrix = 256 × 246, flip angle = 9°, slice thickness = 1.0 mm, voxel size = 1 × 1 × 1 mm^3^). Conventional 2D T1 and T2 images were also acquired for any incidental findings. A diffusion tensor imaging (DTI) pulse sequence with single shot diffusion-weighted echo planar imaging (TR/TE = 6400/95 ms, FOV = 22 × 22 cm^2^, flip angle = 90°, average = 1, matrix = 128 × 128, slice thickness = 3 mm) was applied sequentially in 30 non-collinear directions (b-value = 1000 s/mm^2^). The data analysis was conducted by two researchers who were blind to the status of the subjects.

### SIENA and SIENX analysis

SIENA (http://www.fmrib.ox.ac.uk/fsl) was used to estimate two time-point PBVC and percentage ventricle volume change. Non-brain tissue was removed from all T1-weighted images through the combination of manual and automatic processing. For each subject, the two brain images obtained from the two time-points were first aligned to each other (using the skull images to constrain the registration scaling), with both brain images resampled into the space half-way between the two. Secondly, tissue-type segmentation was carried out to find brain/non-brain edge points and then perpendicular edge displacement (between the two time-points) was estimated at these edge points. Finally, the mean edge displacement was converted into a (global) estimate of PBVC between the two time-points. NBV was estimated with SIENAX (http://www.fmrib.ox.ac.uk/fsl). After brain extraction, the brain images were affine-registered to MNI152 space; this was done primarily in order to obtain the volumetric scaling factor, to be used as a normalization for head size. Tissue-type segmentation with partial volume estimation was then carried out in order to calculate total volume of the brain tissue. The NBV can be optionally split into GMV and WMV. A paired samples t-test was performed to detect global brain volume difference between the two time-points. The statistical parametric map was generated at *p* < 0.05 (Threshold-free cluster enhancement corrected for multiple comparisons).

### FreeSurfer analysis

FreeSurfer (version 5.1.0; http://surfer.nmr.mgh.harvard.edu/) was used for cortical: thickness, surface area, and curvature analysis. The processing stream consisted of the removal of non-brain tissue, mapping to Talairach-like space, and segmentation of the gray-white matter and pial boundaries. These maps of measurements were obtained by reconstructing representations of the GM/WM boundary and the WM boundary to the GM/cerebrospinal fluid boundary, and then calculating the closest distance from those surfaces to each vertex on the tessellated surfaces. All subjects’ data were resampled to the FreeSurfer default common surface template using a high-resolution surface-based averaging technique that aligned cortical folding patterns. Finally, the surface data were spatially smoothed using a Gaussian kernel of 10 mm full-width at half-maximum. Regional variations of the cortical thickness, cortical surface area, and cortical curvature were compared using paired samples t-test. The statistical parametric map was generated at *p* < 0.05 (FDR corrected for multiple comparisons).

### TBSS analysis

Diffusion-tensor images were processed using the FSL 5.0.7 software package (http://www.fmrib.ox.ac.uk/fsl/). The images were corrected for head movement and eddy currents by applying affine alignment of each diffusion-tensor image to the b0 image, and then the b0 image was used to generate a binary brain mask with the Brain Extraction Tool. Subsequently, images were analyzed with the FMRIB’s Diffusion Toolbox (FDT) to generate FA, MD, AD, and RD maps. Statistics on FA maps were performed using the TBSS package in FSL. To create a fractional anisotropy skeleton, the FA images of all subjects were aligned to a template, which was arbitrarily selected from those FA images by nonlinear registrations, and these aligned FA images were transformed to the 1 × 1 × 1 mm MNI152 space. The mean fractional anisotropy skeleton was then thresholded to FA of ≥0.2 to exclude peripheral and intersecting tracts, and the possibility of partial volume effects. Following these steps, individual FA was projected onto mean FA skeleton. The MD, AD, and RD images were analyzed using the FA images to achieve the nonlinear registration and skeletonization stages and also to estimate the MD, AD, and RD images from each individual subject onto the mean FA skeleton. Finally, cross-subject, voxel-wise, statistical analyses of FA, MD, AD, and RD were carried out. In all cases, the null distribution was built up over 5000 permutations by the FSL randomize program. Paired samples t-tests were performed to examine between-test differences. The statistical parametric map was generated at *p* < 0.001 (Threshold-free cluster enhancement corrected for multiple comparisons across space).

### Correlation analyses of brain structures with physiological variables

Pearson correlations were used to assess the correlations of regional cortical thickness and surface area values with body temperature, SaO_2_, and pulmonary variables. Statistical significance was set at *p* < 0.05.

## Additional Information

**How to cite this article**: Fan, C. *et al*. Reversible Brain Abnormalities in People Without Signs of Mountain Sickness During High-Altitude Exposure. *Sci. Rep.* **6**, 33596; doi: 10.1038/srep33596 (2016).

## Figures and Tables

**Table 1:** Physiological characteristics of subjects tested before (Test 1), during (Test 2), and two months after high altitude exposure (Test 3).

	Test 1	Test 2	Test 3	*p*1	*p*2
Heart rate (beats/min)	70.2(10.2)	85.6(13.9)	70.3(13.3)	<0.001	0.964
SaO_2_ (%)	98.3(0.7)	90.5(2.9)	98.2(0.6)	<0.001	0.727
Respiratory rate (breaths/min)	16.8(3.8)	16.0(2.5)	17.5(3.4)	0.337	0.426
Erythrocyte (10^12^/l)	4.7(0.4)	5.2(0.5)	4.8(0.4)	<0.001	0.655
Hemoglobin (g/l)	142.1(12.4)	164.1(18.3)	145.9(13.0)	<0.001	0.252
Blood pressure (mmHg)					
Systolic pressure	112.6(15.5)	118.7(14.5)	113.9(11.3)	0.116	0.720
Diastolic pressure	63.1(9.1)	78.5(10.5)	66.5(9.1)	<0.001	0.146
Pulmonary functions					
FVC (ml)	3661.1(692.3)	2964.4(561.4)	3826.5(450.0)	<0.001	0.216
FEV1 (ml)	3316.2(556.4)	2964.5(486.2)	3419.1(429.9)	<0.001	0.222
FEV1/FVC (%)	91.1(6.1)	94.1(6.6)	89.3(8.3)	0.070	0.360
VC (ml)	3457.1(886.1)	3548.6(821.9)	3533.0(782.3)	0.675	0.21

FVC, forced vital capacity; FEV1, forced expired volume in one second; SaO_2_, arterial oxygen saturation; VC, vital capacity. Data are presented as mean (SD). *p*1: Test 2 vs. Test 1; *p*2: Test 3 vs. Test 1.

**Table 2:** Neuropsychiatric characteristics of subjects tested before (Test 1), during (Test 2), and two months after high altitude exposure (Test 3).

	Test 1	Test 2	Test 3	*p*1	*p*2
Wechsler Memory Scale tests					
1 → 100	59.9(5.9)	62.4(4.0)	60.5(5.3)	0.058	0.702
100 → 1	222.6(11.1)	225.9(8.3)	225.3(8.7)	0.198	0.310
Accumulation	100.2(5.8)	103.1(3.4)	100.1(7.4)	0.020	0.968
Figure memory	16.5(1.9)	18.3(1.5)	17. 7(3.5)	<0.001	0.144
Figure recognition	14.1(1.4)	15.1(1.3)	15.5(0.7)	0.005	<0.001
Figure construction	13.4(1.1)	13.5(0.6)	13.8(0.4)	0.784	0.065
Touch score	418.9(102.1)	470.7(102.5)	495.7(92.1)	0.051	0.003
Digit Span					
Forward task	10.0(0.8)	10.3(0.6)	10.1(0.7)	0.084	0.617
Backward task	8.0(0.9)	8.5(0.7)	8.4(0.7)	0.037	0.048
Rey-Osterrieth Complex Figure test					
Immediate memory	27.8(5.0)	32.3(3.5)	33.3(3.7)	<0.001	<0.001
Long-term memory	28.1(5.1)	32.7(3.1)	33.2(3.6)	<0.001	<0.001
Purdue Pegboard Tests					
Left hand	9.3(1.6)	9.2(1.7)	8.9(1.2)	0.809	0.345
Right hand	10.4(1.1)	10.5(1.4)	10.2(1.1)	0.673	0.785
Both hand	13.8(3.0)	14.0(2.5)	14.2(1.1)	0.732	0.569
Assembly	29.3(6.1)	30.7(5.0)	32.2(5.7)	0.367	0.054
Beck Anxiety Inventory score	25.1(3.5)	23.7(2.3)	23.6(3.3)	0.077	0.093
Beck Depression Inventory score	5.0(3.8)	3.0(3.9)	3.5(3.0)	0.051	0.111

Data are presented as mean (SD).

*p*1: Test 2 vs. Test 1; *p*2: Test 3 vs. Test 1.

**Table 3:** Regional information of changed cortical thickness (mean(SD)) in subjects tested before (Test 1) and during high altitude exposure (Test 2).

Areas	Size (mm^2^)	Test1 (mm)	Test2 (mm)	Tal coordinate			*p* (peak vertex)
				x	y	z	
**Left side**							
Superior frontal gyrus	12522	2.73(0.22)	2.46(0.21)	−11.5	−7.7	47.3	<0.001
Anterior cingulate gyrus	1154	2.94(0.17)	2.50(0.16)	−11.5	40.8	6.0	<0.001
Pericalcarine gyrus	4282	1.45(0.12)	1.70(0.20)	−13.4	−91.2	3.8	<0.001
Fusiform gyrus	2645	2.79(0.43)	2.41(0.40)	−35.1	−23.3	−22.8	0.001
Supramarginal gyrus	1606	2.74(0.24)	2.49(0.23)	−49.6	−48.0	44.6	<0.001
Insula	1446	3.86(0.33)	3.34(0.54)	−35.6	−3.3	−5.5	<0.001
Superior parietal gyrus	1243	2.58(0.31)	2.34(0.26)	−16.2	−66.7	59.2	0.002
Precentral gyrus	516	1.50(0.12)	1.62(0.17)	−38.7	−27.4	49.1	0.003
**Right side**							
Superior frontal gyrus	15595	2.73(0.36)	2.49(0.33)	13.8	22.4	30.5	0.008
Insula	1153	2.88(0.16)	2.61(0.17)	35.4	−5.4	13.3	<0.001
Pericalcarine gyrus	4259	1.61(0.15)	1.93(0.15)	14.3	−77.4	4.8	<0.001
Superior parietal gyrus	583	2.49(0.25)	2.29(0.24)	17.2	−59.4	63.4	0.002
Precentral gyrus	465	1.71(0.16)	1.98(0.16)	33.6	−26.2	45.9	<0.001
Inferior parietal gyrus	459	2.81(0.23)	2.59(0.17)	36.1	−75.9	38.8	<0.001
Supramarginal gyrus	449	3.12(0.30)	2.84(0.20)	57.6	−31.4	44.4	<0.001
Anterior cingulate gyrus	397	2.63(0.28)	2.31(0.18)	8.4	36.0	−5.3	<0.001
